# Limelight on two HIV/SIV accessory proteins in macrophage infection: Is Vpx overshadowing Vpr?

**DOI:** 10.1186/1742-4690-7-35

**Published:** 2010-04-09

**Authors:** Diana Ayinde, Claire Maudet, Catherine Transy, Florence Margottin-Goguet

**Affiliations:** 1 Institut Cochin, Université Paris Descartes, CNRS (UMR 8104), Paris, France; 2 Inserm, U567, 27 rue du faubourg St Jacques 75014 Paris, France

## Abstract

HIV viruses encode a set of accessory proteins, which are important determinants of virulence due to their ability to manipulate the host cell physiology for the benefit of the virus. Although these viral proteins are dispensable for viral growth in many *in vitro *cell culture systems, they influence the efficiency of viral replication in certain cell types. Macrophages are early targets of HIV infection which play a major role in viral dissemination and persistence in the organism. This review focuses on two HIV accessory proteins whose functions might be more specifically related to macrophage infection: Vpr, which is conserved across primate lentiviruses including HIV-1 and HIV-2, and Vpx, a protein genetically related to Vpr, which is unique to HIV-2 and a subset of simian lentiviruses. Recent studies suggest that both Vpr and Vpx exploit the host ubiquitination machinery in order to inactivate specific cellular proteins. We review here why it remains difficult to decipher the role of Vpr in macrophage infection by HIV-1 and how recent data underscore the ability of Vpx to antagonize a restriction factor which counteracts synthesis of viral DNA in monocytic cells.

## Introduction

The monocyte-macrophage lineage represents the only natural target common to all known lentiviruses, which include the primate immunodeficiency viruses (HIVs and SIVs). As a terminally differentiated cell, the macrophage is in a non-proliferative state. The ability to infect non-dividing cells is a characteristic that is unique to members of the lentiviral genus in the retrovirus family (reviewed in [1]). It has opened a promising path in the field of gene therapy since the use of HIV-derived lentiviral vectors has dramatically expanded the range of cell types amenable to gene transfer. The consequence of this property is of course much less fortunate in the setting of natural infection since macrophages are considered major actors in HIV pathogenesis [2-4]. Macrophages are widely recognized as early targets of infection during HIV transmission. They represent long-term producers of the virus due to their higher resistance to HIV cytopathic effects and their relatively long half-life as compared to activated T cells [5]. Macrophages are also capable of migrating into many tissues including the brain. Altogether, these features greatly contribute to transmission, persistence and dissemination of HIV. Accordingly, the multiple aspects of the relationship between HIV and macrophages, as well as other monocytic cells, have long been actively studied.

**Figure 1:**
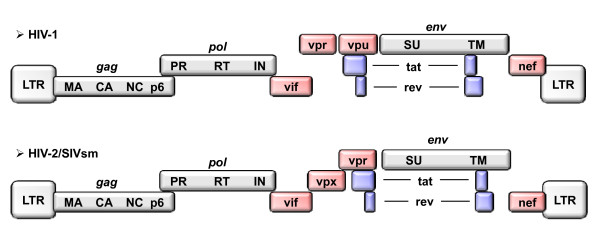
**Schematic representation of HIV-1 and HIV-2 genomes**. Grey boxes represent structural genes; blue boxes indicate regulatory genes; and pink boxes indicate accessory genes.

Sequencing of the genomes of HIV-1 and HIV-2 following their identification as causative agents of human AIDS revealed an unsuspected genetic complexity. In addition to the *gag*, *pol *and *env *genes, which represent the prototypical retroviral genes, the HIV genomes encode two critical regulatory proteins, Tat and Rev, as well as a set of so-called accessory proteins (Figure 1). The latter were soon found to be dispensable for viral growth, at least in certain *ex vivo *cellular systems, hence the term "accessory". However, the emergence of these accessory proteins, which have no counterparts in other retroviral groups, strongly suggested that they fulfill specific needs of HIVs in the context of natural infection [6]. Accordingly, they have stimulated intense investigation for more than 20 years. Here we will focus on the two accessory proteins thought to be more specifically dedicated to macrophage infection by HIVs, namely Vpr, which is shared by HIV-1 and HIV-2, and Vpx, which is specific for the HIV-2/SIVsm lineage. A more general overview of HIV accessory proteins can be found in two recent reviews [7,8]. The unifying view that is currently emerging in this field deserves to be mentioned because it illustrates the quite common necessity for viruses to cope with a cellular environment that may restrict their replication in many ways. Thus, it appears that HIV accessory proteins mainly serve as a means to neutralize host factors that compromise the efficiency of viral replication in particular settings. Moreover, several of these host factors are not passive barriers but rather intrinsic antiviral cell defenses acquired during evolution.

### A common genetic origin for Vpr and Vpx

As mentioned above, the genome of the HIV-2 lineage encodes Vpr, an ortholog of the HIV-1 Vpr protein, and Vpx, which has no counterpart in the HIV-1 lineage [9-11]. Thorough comparisons soon revealed sequence similarity between Vpx and Vpr, pointing to a common ancestral origin of the two genes [12-14]. This similarity extended to an important biological aspect: Vpx and Vpr were shown to be actively encapsidated through their association with the p6 gag product, a feature unique to these two accessory proteins [15-21]. Thus, these proteins are delivered into the cell upon virion entry, suggesting a role in the early steps of the viral life cycle which culminate in the integration of the viral cDNA into the host genome.

It is now widely accepted that HIV-1 and HIV-2 emerged from cross-species transmission of primate lentiviruses that naturally infect chimpanzees (SIVcpz) and sooty mangabeys (SIVsm) respectively [22]. Transmission events from SIVsm-infected mangabeys to macaques also occurred in captive animals giving rise to SIVmac, which causes a disease similar to human AIDS and is therefore largely used as an experimental model [23].

Besides the HIV-1 and the HIV-2/SIVsm lineages, several other lentiviral lineages have been identified in diverse African primates, prompting additional genetic comparisons and phylogenetic studies. The Vpr/Vpx precursor gene is believed to have undergone complex duplication and recombination events during the diversification of primate lentiviruses [14].

Both SIVrcm and SIVmnd2, which infect red-capped mangabeys and mandrils respectively, carry two genes which are likely orthologs of the Vpr and Vpx genes found in the HIV-2 lineage and which have therefore been named Vpr and Vpx [24,25]. All other primate lentiviral lineages, such as African green monkey SIVagm, contain a single gene named Vpr, in reference to the genetic organization of HIV-1 which contains only one of the two genes. However, this nomenclature might be misleading since the corresponding genes do not form a monophyletic cluster and show substantial sequence divergence from the prototypical HIV-1 Vpr [25].

### Role and importance of Vpr in macrophage infection by HIV viruses: absence of a clear picture

Up until quite recently, Vpr had been presented in most publications and reviews as a protein which facilitates HIV-1 macrophage infection by contributing to nuclear import of the viral pre-integration complex (PIC) [26]. Several lines of evidence supported this view. In the retrovirus life cycle, integration of viral cDNA into the host DNA is a prerequisite for the synthesis of new virions. Unlike gammaretroviruses, lentiviruses can achieve this step independently of the nuclear envelope breakdown which occurs during mitosis, hence their ability to infect non-cycling cells. This property implies that lentiviruses such as HIVs have evolved a mechanism that enables efficient nuclear translocation of the viral PIC which is formed in the cytoplasm after completion of the reverse transcription step. As a virion-packaged protein, Vpr is present from the start of the virus life cycle and based on their finding that it also displays nucleophilic properties, Lu et al. hypothesized that Vpr might contribute to nuclear targeting of the viral PIC [27]. A number of studies provided experimental evidence consistent with this prediction although pointing to functional redundancy between Vpr and other karyophilic viral components such as the Matrix protein [28-34]. Moreover, it was reported that Vpr-deficient HIV-1 showed a significant replication defect in primary macrophages but not in established cell lines or activated T cells [35,36]. Thus, Vpr seemed to be required for optimal viral replication in non-cycling target cells.

However, later studies showed that HIV infection of non-cycling cells does not depend on the karyophilic properties of viral proteins [37-42]. In addition, HIV-1-derived lentiviral vectors devoid of accessory proteins efficiently transduce terminally differentiated cells such as neurons [43-45]. Thus, the ability of Vpr to enhance infection of macrophages has to be related to specific features of these cells rather than to their non-cycling status. In further support of this hypothesis, experiments conducted using lymphoid tissue explants showed that tissue macrophages were less permissive to infection by Vpr-deficient HIV-1 than by wild type HIV-1, whereas resting T cells within the same tissue were equally well infected by both types of viruses [46]. Therefore, it remains unclear whether a helper role in nuclear import of viral DNA represents the functional basis for Vpr-mediated increase in macrophage infection by HIV-1. It should also be noted that a role of Vpr in the nuclear targeting of viral DNA would rely on the Vpr pool present in the incoming virion, which was shown to be much less abundant than initially estimated [47].

It is somewhat difficult to estimate the extent of the facilitation effect of Vpr in macrophage infection by HIV-1. In single cycle infection experiments, the absence of Vpr resulted in only a two-fold decrease in the percentage of transduced macrophages [43]. Although modest, this effect on transduction efficiency is expected to be cumulative in a spreading infection using replicative viruses. This may partly explain the much larger defect range (> 10-fold) reported in the two studies which are commonly taken as reference [35,36]. Of note, in other studies, which also used propagative viruses, the lack of Vpr had a lower effect on viral replication [28,48]. If *de novo *synthesized Vpr contributes to the spreading of infection in macrophages, as was proposed by Connor et al [36], it might also amplify the quantitative differences between single cycle and spreading infections. In that case, the overall effect of Vpr would result from two distinct Vpr functions, the first occurring prior to viral integration and performed by the virion-delivered Vpr pool, the second taking place after viral integration and performed by *de novo *synthesized Vpr. Consistent with a function during the late steps of the viral life cycle, Vpr has been shown to stimulate HIV-1 transcription in monocytic cells [49]. Strong variations between donors were also recurrently reported regarding the effect of Vpr on viral replication in macrophages [35,50], adding another difficulty in interpreting the data obtained in these experimental systems. Moreover, the magnitude of Vpr's effect appears to depend on the multiplicity of infection used in the initial inoculum [51].

Although no systematic mutagenesis approach has been carried out in order to genetically dissect the helper effect of Vpr in the context of macrophage infection, several studies have addressed whether this effect segregates with another property of the protein. Remarkably, reduced viral replication in macrophages was observed for mutations of Vpr disrupting its interaction with host proteins as diverse as uracil-DNA glycosylase, hCG1 nucleoporin, importin alpha and exportin 1 [50,52-54]. Thus to date, no unifying view has emerged regarding the putative host partners of Vpr in its ability to increase the replicative capacity of HIV-1 in macrophages.

Finally, the functional importance of Vpr in macrophage infection markedly differs between lentiviral lineages. As described above, HIV-1 Vpr appears to facilitate macrophage infection but is not critical. HIV-2/SIVsm replication was found to be only mildly - if at all - affected by the lack of Vpr [55-58], whereas SIVagm was reported to be critically dependent on Vpr for macrophage infection [59]. The fact that Vpr knock-out shows such species-specific effects with respect to macrophage infection suggests that the Vpr genes have significantly diverged from each other in the selective advantages they confer to their cognate viruses. This also indicates, as mentioned previously, that they are not genuine orthologs.

### A potential link between Vpr and HIV evasion of host immune defenses

Macrophages and dendritic cells (DC), in addition to being targets and reservoirs for HIV, play a critical role in the coordination between innate and adaptive immune responses to infection. Following detection and capture of pathogen products, these cells undergo a maturation process and deliver activation signals to antigen-specific T cells through both cell-cell contacts and secretion of cytokines. In turn, activated T cells secrete specific cytokines which potentiate both the adaptive and innate immune responses. There is increasing evidence that these complex regulatory circuits are impaired by persistent viruses such as HIVs, as a means to undermine the overall host antiviral immune response [60]. The possible contribution of Vpr to HIV-1 immune escape was addressed by several groups. Two reports showed that Vpr downregulates the expression of Interferon regulatory factor 3 (IRF-3) [61,62], a factor which is essential for interferon (IFN) beta production in response to viral infection. The lack of IRF-3 activation during the course of HIV-1 infection is however unrelated to Vpr's effect since deletion of Vpr did not restore IFN beta production by HIV-1-infected cells [62]. A remarkably broad spectrum of Vpr-mediated immunosuppressive effects was documented in immune cells. Thus, Vpr was found to impair DC/macrophage maturation, to compromise natural killer (NK) effector functions, to induce apoptosis of cytotoxic T cells, to downregulate the production of chemokines by macrophages and T cells, and to compromise T cell activation pathways [63-70]. Overall, Vpr appears to impair the so-called Th1 immune response, known to be critical for antiviral immunity (reviewed in ref [71]). This raises the interesting possibility that Vpr contributes to viral persistence by compromising the proper cooperation between immune cells rather than by (or in addition to) modulating the viral replicative capacity at the cell level. However, there are unsolved questions and controversies. For example, several of the immunomodulatory effects of Vpr were observed using cells exposed to extracellular recombinant Vpr protein and whether this unusual way of delivering Vpr is biologically relevant to the setting of natural infection requires confirmation. Conflicting data have been reported regarding the effect of Vpr on NK cells. In two studies, Vpr induced anergy of bystander NK cells [69,70], whereas in another study, Vpr stimulated their ability to recognize and lyse infected cells [72]. How Vpr might alter immune functions of infected or bystander cells is unclear as well. It has been proposed that Vpr acts by enhancing the immunosuppressive action of endogenous glucocorticoids [73] or by inhibiting the activity of NFkB, a key transcription factor in the induction of proinflammatory mediators [68]. However, opposite effects of Vpr on NFkB were reported by other investigators [49,74]. Mutations which abolish the immunosuppressive activities of Vpr have not yet been characterized. Finally, it will be important to address whether presumed orthologs of HIV-1 Vpr also show immunoregulatory properties, given that persistent infection is a hallmark of primate lentiviruses.

### Mechanism of Vpr-mediated cell cycle arrest: a clue for its actual function?

Expression of Vpr in cycling cells triggers cell cycle arrest at the G2 phase, i.e. after completion of DNA replication and prior to mitosis. Vpr-mediated G2 arrest, which was underscored in 1995 by several groups [75-79], represents by far the most widely admitted property of the viral protein. However, the biological significance of this activity is unclear given that efficient viral replication does not require Vpr in dividing cells infected *ex vivo*. Conversely, Vpr-mediated G2 arrest is obviously irrelevant to its elusive role in HIV-1 infection of macrophages since these cells do not divide.

**Figure 2:**
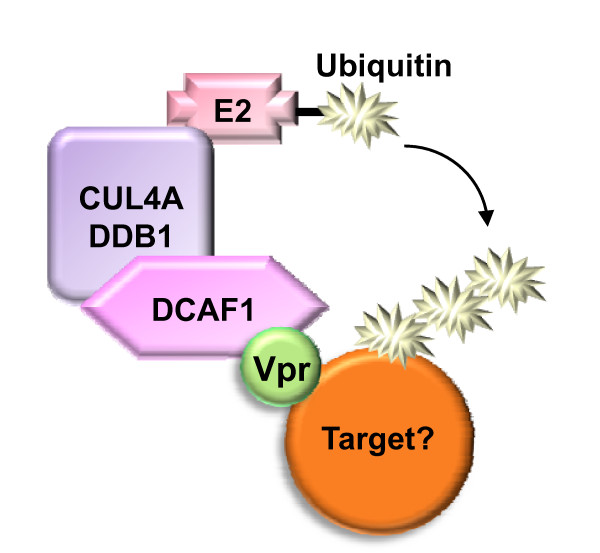
**Model for HIV-1 Vpr mechanism of action**. HIV-1 Vpr recruits the Cul4A-DDB1 ubiquitin ligase through DCAF1 binding, which leads to the ubiquitination and inactivation of an unknown cellular target required for entry into mitosis.

In spite of the above paradox, the underlying mechanism of Vpr-mediated G2 arrest has been searched for during many years. In 2007, these efforts finally bore fruit; when several studies, including ours, identified VprBP/DCAF1 as a critical host factor in the ability of Vpr to promote cell cycle arrest [80-86]. Zhao et al. had previously identified VprBP as a cellular protein binding to Vpr with high affinity [87]. Unfortunately, at the time, this finding had not drawn much attention presumably because of the plethora of proposed Vpr host partners. More recent studies in a totally different field crossed the path of VprBP, which was isolated as a member of the DDB1-Cullin4-associated factors (DCAFs) [88-91]. DDB1 represents a core component of complexes assembled by Cullin 4 (Cul4), which act as E3 ubiquitin ligases. These cellular enzymes control the selection of protein substrates that will undergo ubiquitin conjugation, a prerequisite for their subsequent proteasome-mediated degradation. In a given Cul4-based complex, substrate specificity is likely dictated by the nature of the DCAF protein bound to DBB1. Of note, while investigating another activity of Vpr, Landau's group reported the association of Vpr with two members of the cullin-based ubiquitin ligase family Cullin 1 and Cullin 4 [92]. Regarding the physical and functional connection between Vpr and VprBP, now renamed DCAF1, the data collectively obtained by the different investigators support the model depicted in Figure 2[80-85]. Vpr simultaneously recruits the Cul4A ubiquitin ligase through DCAF1 and a so far unknown cellular protein which is required for entry into mitosis. As a result, the Vpr target protein undergoes ubiquitination and subsequent proteasome-mediated degradation, which in turn precludes the G2/M transition of the cell cycle. Vpr itself appears to be immune to DCAF1-induced degradation and is even stabilized by its association with the Cul4A-DDB1 ubiquitin ligase [93].

Diverting the host ubiquitination machinery to inactivate an undesirable host protein is a fairly common viral strategy. Two HIV-1 accessory proteins have previously been shown to use this mechanism. Vpu promotes destabilization of the intracellular pool of CD4 [94], to ensure the liberation of infectious viruses, and inactivates BST-2/tetherin, a cellular membrane protein which traps newly synthesized virions at the cell surface [95-97]. Vif induces the degradation of the antiviral APOBEC3G and APOBEC3F enzymes, preventing their incorporation into newly formed virions and therefore avoiding their deleterious effects during the following infection cycle [98-101].

It should be stressed that the model proposed for the Vpr mechanism of action does not enlighten us about the biological significance of G2 arrest in the context of viral infection. As already mentioned, Vpr does not confer a measurable growth advantage to HIV in the dividing cells used for *ex vivo *infection experiments. However, both the transcription and the translation of viral genes reach maximal levels during the G2/M phase of cell cycle and it has been suggested that in the context of natural infection, Vpr-mediated G2 arrest could serve as a means to optimize viral production by the short-lived infected cells [102,103]. In view of our present knowledge of Vpr's mechanism of action, one might also consider that the intended effect of Vpr is the degradation of a specific host protein rather than the resulting cell cycle arrest. In this case, the Vpr host target is expected to be detrimental for viral replication or persistence in the context of natural infection in vivo. Identification of the host protein(s) targeted for degradation by Vpr will certainly be instrumental for our understanding of the genuine function of this accessory protein.

Vpr from the SIVsm/HIV-2 lineage also binds DCAF1 [82,86] and triggers G2 arrest albeit less efficiently than HIV-1 Vpr [56,86,104-107]. This strongly suggests that the Vpr proteins from HIV-1 and HIV-2 use the same mechanism of action and target at least one common host protein. The latter is, however, unlikely to play a role in the relationship between HIVs and macrophages given that Vpr-deficient HIV-2 efficiently replicate in these cells. That an additional factor is inactivated by HIV-1 Vpr to facilitate macrophage infection might, however, be envisioned in light of the recently revealed ability of Vpr to divert the host ubiquitination machinery.

### An earlier view of Vpx as the functional counterpart of HIV-1 Vpr in macrophage infection

As previously mentioned, members of the HIV-2/SIVsm lineage carry both Vpr and Vpx, the latter being specific to this lineage (Figure 1). The common genetic origin of Vpr and Vpx raised the question of their respective functions in the HIV-2 lineage as compared to that of the single Vpr protein encoded by HIV-1.

Early data showed that Vpr from HIV-2/SIVsm, like Vpr from HIV-1, had the ability to arrest the cell cycle at the G2 phase whereas Vpx had no effect on cell cycle progression [56,104-106]. However, several lines of evidence supported a role for Vpx in the nuclear import of viral DNA in non-dividing cells. First, a number of studies reported that Vpx is critical for the infection of non-dividing macrophages, but not of cycling cells [56,108,109]. Secondly, the replication defect induced by Vpx mutants correlated with the failure to promote accumulation of 2LTR circles, which are considered as markers of viral DNA entry into the nucleus. Finally, the nuclear localization of Vpx in macrophages was consistent with its ability to promote productive infection [110-113]. Therefore, it was hypothesized that the two activities attributed to HIV-1 Vpr had somehow segregated in the HIV-2/SIVsm lineage: the ability to induce G2 arrest became specific to Vpr in this lineage, and the ability to import viral DNA into the nucleus became specific to Vpx [56]. However, uncertainties remained regarding the role of Vpx in viral DNA nuclear import. Some studies reported a role of Vpx in the infection of dividing T cells in addition to its role in macrophages [57,58,114-118], while other studies failed to detect a nuclear localization of Vpx [119]. Of note, Vpx was also found to interfere with the reverse transcription step in early studies. However, this effect appeared minor compared to the effect on the viral DNA nuclear import and therefore was not further investigated [56].

### First evidence that Vpx is not the functional counterpart of HIV-1 Vpr in macrophage infection

Although the deletion of Vpr in HIV-1 induces a moderate decrease of HIV-1 transduction in macrophages, its effect was minor compared to that of Vpx in SIV/HIV-2 [58,112,115,118,120]. The requirement for Vpx is even stronger in monocytes and in monocyte-derived dendritic cells infected by SIVmac, in which cell transduction is entirely dependent on its presence [121-124]. These data underscore the major role of Vpx during the infection of cells from the monocytic lineage. As already mentioned, the picture is not very clear in lymphoid cells where the dependence on Vpx for viral growth is still controversial.

*In vivo *experiments in macaque models also pointed out major differences between viruses deleted for Vpr or Vpx [125,126]. Surprisingly, in the two studies, Vpr-deleted viruses behaved similarly to the wt viruses. In experiments using the acutely pathogenic virus SIVsmPBj inoculated into pigtailed macaques, the effect of Vpx deletion is dramatic: macaques infected with the wt virus developed fulminant disease whereas animals inoculated with the Vpx-deleted virus showed delayed kinetics of viral replication and no disease manifestations [126]. In the study using rhesus monkeys infected with a virus derived from SIVmac239, progression to death occurred in the absence of a gene for Vpx, but lower virus burdens and delayed disease induction were noticed [125].

The essential role of Vpx both in cells from the monocytic lineage and *in vivo *suggests that Vpx is not just a pale imitation of Vpr in its ability to transport the viral DNA.

### A specific function for Vpx: degradation of a cellular restriction factor in macrophages

It came as a surprise when Vpx was found to promote the accumulation of HIV-2/SIV reverse transcripts and therefore to act prior to the transport of the pre-integration complex. These findings were first demonstrated in monocyte-derived dendritic cells and thereafter in macrophages [55,122,127-129]. Whether Vpx plays a role in nuclear import of viral DNA in addition to its role at the reverse transcription step remains a subject of debate [109].

**Figure 3:**
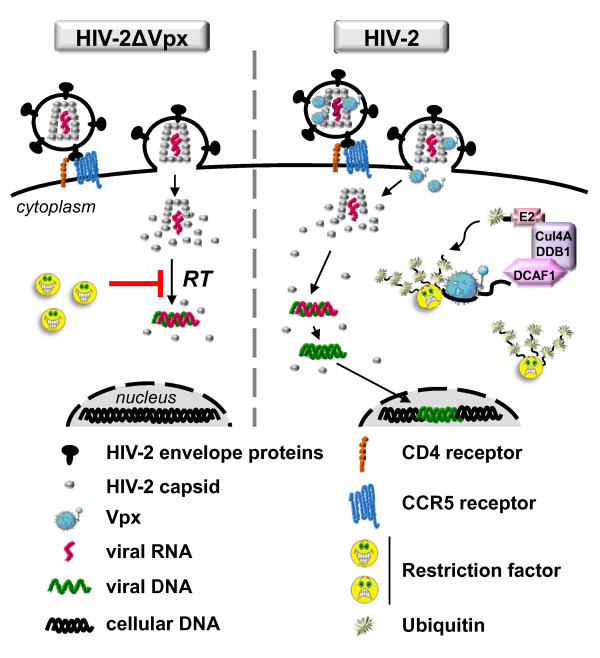
**Vpx recruits the Cul4A-DDB1^DCAF1 ^ubiquitin ligase to counteract a cellular retriction in the early phases of macrophage infection**. Vpx is absolutely required for HIV-2 infection of human monocyte-derived macrophages. During the early steps of replication, Vpx, a virion-packaged protein, hijacks the Cul4A-DDB1 ubiquitin ligase by binding to DCAF1. This leads to the inactivation of a macrophage-specific restriction factor, resulting in the completion of reverse transcription and the integration of viral DNA into the host chromosome. In the absence of Vpx, the restriction factor blocks the accumulation of HIV-2 reverse transcripts.

It appeared unlikely that Vpx targeted HIV and SIV viral components since the effect of Vpx, which is particularly dramatic in dendritic cells, appeared to be cell-type dependent and since Vpx facilitated viral transduction of dendritic cells with retroviruses as divergent as primate lentiviruses (HIV-1), non-primate lentiviruses (FIV) or gammaretroviruses (murine leukemia viruses) [122]. The question was, did Vpx complement the lack of a cellular activity, such as viral DNA nuclear import, or did Vpx counteract an antiviral activity present in dendritic cells. The ability of the proteasome inhibitor MG132 to partially restore infectivity of Vpx-deficient SIVmac lentiviral particles led Cimarelli's group to propose that Vpx promotes retroviral escape from a proteasome-dependent restriction pathway present in monocyte-derived dendritic cells [122]. Based on sequence homology between Vpr and Vpx, we predicted that Vpx may divert the same ubiquitin ligase as Vpr and we demonstrated, using a two hybrid system that Vpx from SIVsmPBj physically interacts with the DCAF1 adaptor subunit of the Cul4A-DDB1 ubiquitin ligase [83]. Binding of Vpx from SIV or HIV-2 to DCAF1 was further confirmed in mammalian cells [55,129,130]. Altogether, the data pointed to a hypothesis in which Vpx would use the DCAF1 ubiquitin ligase to get rid of a cellular restriction factor present in dendritic cells and in macrophages. This hypothesis received experimental support in macrophages in the context of SIVsmPBj, SIVmac239 and HIV-2 infection [55,129,130]. Stevenson's group demonstrated that Vpx counteracts a macrophage-specific restriction factor using heterokaryons, a technique previously adopted to characterize the mechanism of action of another HIV accessory protein, Vif [131,132]. Heterokaryons generated between COS cells, in which Vpx is dispensable for virus infection, and macrophages, in which Vpx is essential for virus infection, supported transduction by wt SIV but not Vpx-deleted SIV [130]. This definitively discarded the possibility that Vpx may complement the lack of a cellular factor necessary for viral replication in macrophages. Vpx, like Vpr, is a virion-incorporated protein and was therefore expected to perform its function prior to *de novo *synthesis of viral proteins in the target cell. In agreement with this, infection of macrophages harboring wt Vpx alleviated the block to subsequent infection by Vpx-deficient SIV, providing evidence that Vpx delivered *in trans *can counteract the restriction [130]. Two studies showed that inactivation of the DCAF1-binding property of Vpx mimicked the absence of Vpx, impairing HIV-2/SIV growth in macrophages. In addition, silencing of DCAF1 or DDB1 impaired replication of wt HIV-2/SIV [55,129,130]. This latter result argues against a model in which Vpx would inhibit the endogenous activity of the ubiquitin ligase, and subsequently prevent the degradation of a positive factor, but rather favors a model in which Vpx diverts the activity of the Cul4A-DDB1^DCAF1 ^complex to induce the degradation of a macrophage-specific restriction factor (Figure 3).

What properties can be attributed to this restriction factor? We must underline here that the restriction factor targeted by Vpx is unlikely to also be inactivated by HIV-1 Vpr since infectivity of Vpr-deleted HIV-1 is dramatically enhanced in macrophages by Vpx but not by HIV-1 Vpr [7,121,130].

The restriction factor antagonized by Vpx appears to inhibit the accumulation of reverse transcripts, a step which is also targeted by the Trim5α restriction factor [133-137]. However, the two proteins differ in many aspects. In contrast to Trim5α, the restriction factor antagonized by Vpx should be conserved across primate species since SIVsm Vpx can substitute HIV-2 Vpx in HIV-2 macrophage infection and HIV-2 Vpx can complement Vpx-defective SIVmac. Also in contrast to Trim5α, which is ubiquitous, the Vpx-targeted restriction factor is specific of the monocytic lineage. However, unlike Trim5α, this restriction factor is probably not saturable, at least in dendritic cells, since high amounts of viral particles fail to restore infectivity of Vpx-deficient SIVmac [122,123]. Similarly to Trim5α though, the Vpx-antagonized factor is type I IFN-inducible as highlighted lately by a report [7].

Recently, Vpx has been shown to counteract a cellular restriction in monocytes, which are highly susceptible to SIVsmPBj infection provided that Vpx is expressed [124,128,138]. Whether a unique restriction factor is antagonized by Vpx in macrophages, dendritic cells and monocytes remains to be clarified. In the latter, the potential dependence on the Cul4A-DDB1^DCAF1 ^ubiquitin ligase has not been addressed. Surprisingly, a DCAF1-independent role of Vpx has been described in dendritic cells, as well as in differentiated monocytic THP1 cells which are supposed to recapitulate the functional properties of macrophages [121]. The possibility remains that Vpx uses different mechanisms in different cell types to counteract a cellular restriction. It is striking however, that Vpx in both macrophages and dendritic cells acts at the level of reverse transcription. Further investigations will be needed to understand whether the reverse transcription step itself is inhibited by the cellular restriction, whether an abnormal destruction of the newly synthesized viral cDNA occurs, or whether a step prior to the reverse transcription is affected.

The ability of HIV-1 to transduce macrophages, and to a lesser extent dendritic cells, in the absence of any accessory proteins may suggest that the cellular restriction factor antagonized by Vpx senses a viral component that is highly specific for HIV-2/SIVsm and which is not present in HIV-1. However, the restriction antagonized by Vpx is also active against HIV-1 since Vpx increases the permissivity of dendritic cells and macrophages to HIV-1. In addition, monocytes, which are normally refractory to HIV-1, become permissive in the presence of Vpx [121,122,128,130,138-140]. In macrophages, the helper effect of Vpx towards HIV-1 transduction has been shown to depend on DDB1 [130] and thus probably also relies on the hijacking of the Cul4A ubiquitin ligase, leading to subsequent degradation of an inhibitory factor. Whether such a mechanism exists in monocytes has not been addressed yet. If the same restriction factor antagonizes HIV-1 in monocytes and macrophages, its level of expression is expected to be higher in monocytes due to their resistance to HIV-1 infection (c.f. the accompanying review by A. Bergamaschi and G. Pancino).

### Vpr and Vpx: rival brothers?

To date, the only functional characteristic common to Vpr and Vpx is their ability to recruit the Cul4A ubiquitin ligase. This raises the possibility that Vpr and Vpx compete for function. In the early steps of the viral life cycle, such a competition is difficult to envision given that the amount of virion-bound Vpr and Vpx is minor compared to the pool of DCAF1. In addition, it has been shown that deletion of Vpr does not affect Vpx-dependent HIV-2/SIV infection [7,55]. However, in later steps of viral infection, competition might occur assuming that the amount of *de novo *synthesized Vpr and Vpx now exceed that of DCAF1. Consistent with this possibility, G2 arrest-defective mutants of Vpr which conserve DCAF1-binding activity were shown to inhibit G2 arrest induced by wt Vpr, suggesting that the amount of DCAF1 might be limiting [81,83]. Whether this probable competition in turn affects Vpr and Vpx functions requires further investigation.

## Conclusion

The ability to recruit the Cul4A ubiquitin ligase is shared by Vpr and Vpx proteins from diverse lentiviral origins, suggesting that this trait was acquired early in the evolution of the vpr-like gene family. While conserving this functional characteristic, Vpr and Vpx have likely diverged in the nature of the substrates they target and therefore in their respective functions. The modest effect of HIV-1 Vpr deletion on viral replication in macrophages and the absence of a clear phenotype in *ex vivo *cell cultures for Vpr-deleted HIV-1 raised questions regarding the true role of Vpr in viral infection. Further investigation is required to understand whether Vpr plays a role in early steps of infection, as expected considering its virion-bound nature, or whether Vpr plays a more indirect role by regulating the spread and (or) persistence of infection, as would suggest its reported immunomodulatory properties [65,66,69,70]. What is certain is that Vpr must confer some selective advantage to the virus since reversion events in *vpr *occur rapidly in rhesus monkeys infected with Vpr-defective SIVmac [125]. The requirement for the Cul4A-DDB1^DCAF1 ^ubiquitin ligase, which was first shown for Vpr, has not unravelled the role of Vpr-mediated G2 arrest during viral infection. Somewhat ironically, this property has enlightened the underlying mechanism of the well known critical function of Vpx in macrophage infection. It is now widely acknowledged that Vpx specifically counteracts a cellular restriction present in macrophages leading to establishment and productive spread of infection. Why HIV-1 Vpr has not kept or acquired this powerful activity from an ancestral vpr-like gene remains a mystery, in particular since this restriction appears to be active against HIV-1 [122,130]. The discovery of the cellular target(s) of Vpr and Vpx will provide great help in understanding the susceptibility of cells from the monocytic lineage to infection by both HIV-1 and HIV-2/SIVsm. This might in turn lead to the elaboration of antiviral strategies to prevent viruses from establishing reservoirs in these cells.

## Competing interests

The authors declare that they have no competing interests.

## Authors' contributions

CT and FMG conceived and wrote the review. DA and CM conceived the illustrations and were involved in discussions and critical reading of the manuscript.
